# Body Mass Parameters, Lipid Profiles and Protein Contents of Zebrafish Embryos and Effects of 2,4-Dinitrophenol Exposure

**DOI:** 10.1371/journal.pone.0134755

**Published:** 2015-08-20

**Authors:** Nancy Hachicho, Sarah Reithel, Anja Miltner, Hermann J. Heipieper, Eberhard Küster, Till Luckenbach

**Affiliations:** 1 UFZ—Helmholtz Centre for Environmental Research, Department of Environmental Biotechnology, Permoserstraße 15, 04318, Leipzig, Germany; 2 UFZ—Helmholtz Centre for Environmental Research, Department Bioanalytical Ecotoxicology, Permoserstraße 15, 04318, Leipzig, Germany; University of Siena, ITALY

## Abstract

Morphology and physiology of fish embryos undergo dramatic changes during their development until the onset of feeding, supplied only by endogenous yolk reserves. For obtaining an insight how these restructuring processes are reflected by body mass related parameters, dry weights (dw), contents of the elements carbon and nitrogen and lipid and protein levels were quantified in different stages within the first four days of embryo development of the zebrafish (*Danio rerio*). The data show age dependent changes in tissue composition. Dry weights decreased significantly from 79μgdw/egg at 0hours post fertilization (hpf) to 61 μgdw/egg after 96 hpf. The amounts of total carbon fluctuated between 460 mg g^-1^ and 540 mg g^-1^ dw, nitrogen was at about 100 mg g^-1^ dw and total fatty acids were between 48–73 mg g^-1^ dw. In contrast to these parameters that remained relatively constant, the protein content, which was 240 mg g^-1^ at 0 hpf, showed an overall increase of about 40%. Comparisons of intact eggs and dechorionated embryos at stages prior to hatching (24, 30, 48 hpf) showed that the differences seen for dry weight and for carbon and nitrogen contents became smaller at more advanced stages, consistent with transition of material from the chorion to embryo tissue. Further, we determined the effect of 2,4-dinitrophenol at a subacutely toxic concentration (14 μM, LC10) as a model chemical challenge on the examined body mass related parameters. The compound caused significant decreases in phospholipid and glycolipid fatty acid contents along with a decrease in the phospholipid fatty acid unsaturation index. No major changes were observed for the other examined parameters. Lipidomic studies as performed here may thus be useful for determining subacute effects of lipophilic organic compounds on lipid metabolism and on cellular membranes of zebrafish embryos.

## Introduction

The development of embryos involves a continuous, fundamental restructuring of the embryonic organism. Starting point is a single undifferentiated cell that develops into a complex organism with organs and tissues consisting of a vast number of highly differentiated cells. Embryos developing in an egg such as fish embryos do not take up external nutrients but use their yolk supplies for growth, development and as energy source. The overall exchange of ions and water between the embryo tissue and the external medium is kept low by certain structures such as tight junctions between epidermal cells forming a barrier impermeable for many molecules but enabling gas exchange for respiration. In contrast, more advanced fish life stages maintain comparatively high levels of exchange between internal compartments and the exterior via specific organs (gills and intestines) with large surface areas at the organism—environment interfaces for gas and ion exchange and the uptake of nutrients [1].

Regarding their morphology and physiology, early developmental and adult stages of fish substantially differ. This suggests that those stages may be differently affected and thus differently sensitive to toxic agents. However, it has been shown that sensitivities of embryo/larval stages and of adult fish to toxic agents largely correlate and that experimental data obtained with fish embryos can be extrapolated to adult stages in many cases [2,3]. In contrast to further advanced stages, experiments with fish eggs and eleutheroembryos are not regarded as animal experiments in many countries and they are therefore promoted as an alternative ecotoxicological experimental fish model [4].

In recent years, embryos of the zebrafish (*Danio rerio*) have become a widely used model system in (eco)toxicology, disease research, pharmacology, drug development and developmental biology [5–11]. Protocols for acute toxicity tests with zebrafish embryos have been worked out [7,12], and good correlations were found between results obtained with zebrafish embryo and acute adult fish toxicity tests for more than 140 chemicals [2,3,13]. Furthermore, the availability of detailed information regarding genomics, transcriptomics and metabolomics [14–17] makes zebrafish embryos suitable as model system in molecular ecotoxicology studies [10]. Zebrafish embryos have been used in some studies on lipid metabolism performed in the context of disease research [18,19]. However, so far lipids and other physiological parameters and their changes during embryonic development have not been systematically quantified in zebrafish. This information would be particularly useful for the development of physiologically based toxicokinetic (PBTK) models, which are based on knowledge on body composition and physiology [20].

Considering that growth of the fish embryo proceeds without uptake of external resources and thus entirely relies on endogenous supplies, we address the question how the restructuring process during development is reflected by changes in body mass related parameters. We therefore quantified dry weight and tissue contents of the elements carbon and nitrogen as essential components of biological molecules as well as fatty acids and protein as important body composition parameters in different embryonic zebrafish stages up to 96 hours post fertilization (hpf). The major focus of this study was on lipidomics; this included also the optimization of extraction and quantification methods for fatty acids in different lipid classes in zebrafish embryos.

Since the zebrafish embryo is an important experimental model in (eco)toxicology and pharmacology, we examined to which degree these parameters are influenced by exposure to a toxic chemical as this may be important for interpretation of toxic effects. We therefore conducted measurements with embryos that had been exposed to 2,4-dinitrophenol (2,4-DNP), in addition to analyses of unchallenged embryos. 2,4-DNP was chosen as chemical stressor as we expected effects particularly from interference with lipid metabolism. The compound is a classical mitochondrial protonophore shown to disrupt the cellular energy supply [21] and to cause an increase of membrane fluidity by interaction with membrane components [22]. With this experiment we aimed at determining to which degree the lipid fatty acid composition in fish embryos is robust to such chemical challenge and to provide indications for the potential of the embryos for an adaptive response.

## Results

### Dry weight, carbon, nitrogen, protein and fatty acid profiles of different zebrafish embryonic stages

**Fig 1:**
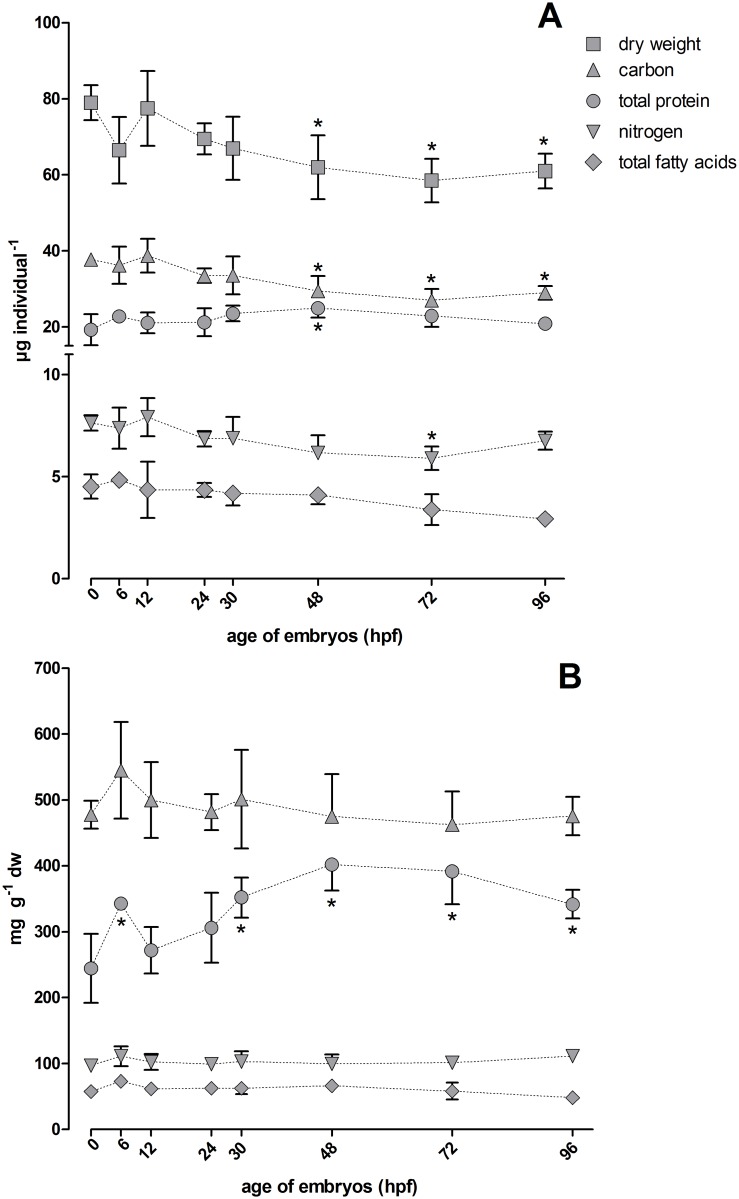
Values for dry weight and for carbon, nitrogen, total fatty acids and protein contents for eggs/hatched embryos of the zebrafish (*D*. *rerio*) at different stages of development. (A) Absolute values for the different parameters per individual and (B) relative amounts of carbon, nitrogen, total fatty acids and protein normalized to respective dry weights are depicted. Symbols were connected to visualize trends. *: p < 0.05 (Dunnett’s test with 0 hpf as reference). For dry weight, carbon and nitrogen: n = 4 with 5 pooled individuals per n; for proteins: n = 5–7 with 30 pooled individuals per n; for fatty acids: n = 3–4 with 50 individuals per n; hpf: hours post fertilization. Note that the y-axis in A is divided into two sections with different scales.

Values for dry weight, the amounts of carbon and nitrogen elements and of proteins and total fatty acids for zebrafish embryonic stages between 0 and 96 hpf are shown in Fig 1.

We use the following terminology for the studied stages: The terms “egg” and “embryo with chorion” denominate the embryo in the chorion prior to hatching; “hatched embryo” or “eleutheroembryo” is used for embryos that have hatched from the chorion; “dechorionated embryo” is the embryo, from which the chorion was manually removed.

The amounts of carbon, nitrogen, proteins and lipids are per single egg or hatched embryo, depending on stage (Fig 1a), or referred to respective dry weights (Fig 1b). Hatching of embryos commences between 48 and 72 hpf and is completed between 72 and 96 hpf, which raises the question whether the loss of the chorion affects these values. As detailed further below (this section, subsection “chorion”), there is evidence that constituents of the chorion become available as physiologically relevant resources to the embryo within the chorion and that the contribution of the chorion to dry weight, carbon and nitrogen content decreases over time. In the overview of body mass parameters in Fig 1 we therefore present the values with chorion (as long as present) for respective embryo stages.

#### Dry weight

Mean dry weights as a measure for total biomass of embryos ranged between 58 and 79 μg per individual at different stages. Dry weights showed a general decrease, which amounted to 23% between 0 and 96 hpf (Fig 1A). The relatively low dry weight at 6 hpf was not statistically significantly different from the value at 0 hpf (p > 0.05, Dunnett’s test with 0 hpf as control group). It may mirror a certain degree of variation in our samples and may not indicate a trend in dry weight change. However, variations of dry weight also affect values of the determined parameters if referred to dry weight. Similar episodic variations in other parameters at 6 hpf will therefore not be regarded as real changes. Dry weight values that were at a similar level at 48–96 hpf were significantly lower than the 0 hpf value (p < 0.05) (Fig 1A).

#### Fatty acids

**Fig 2:**
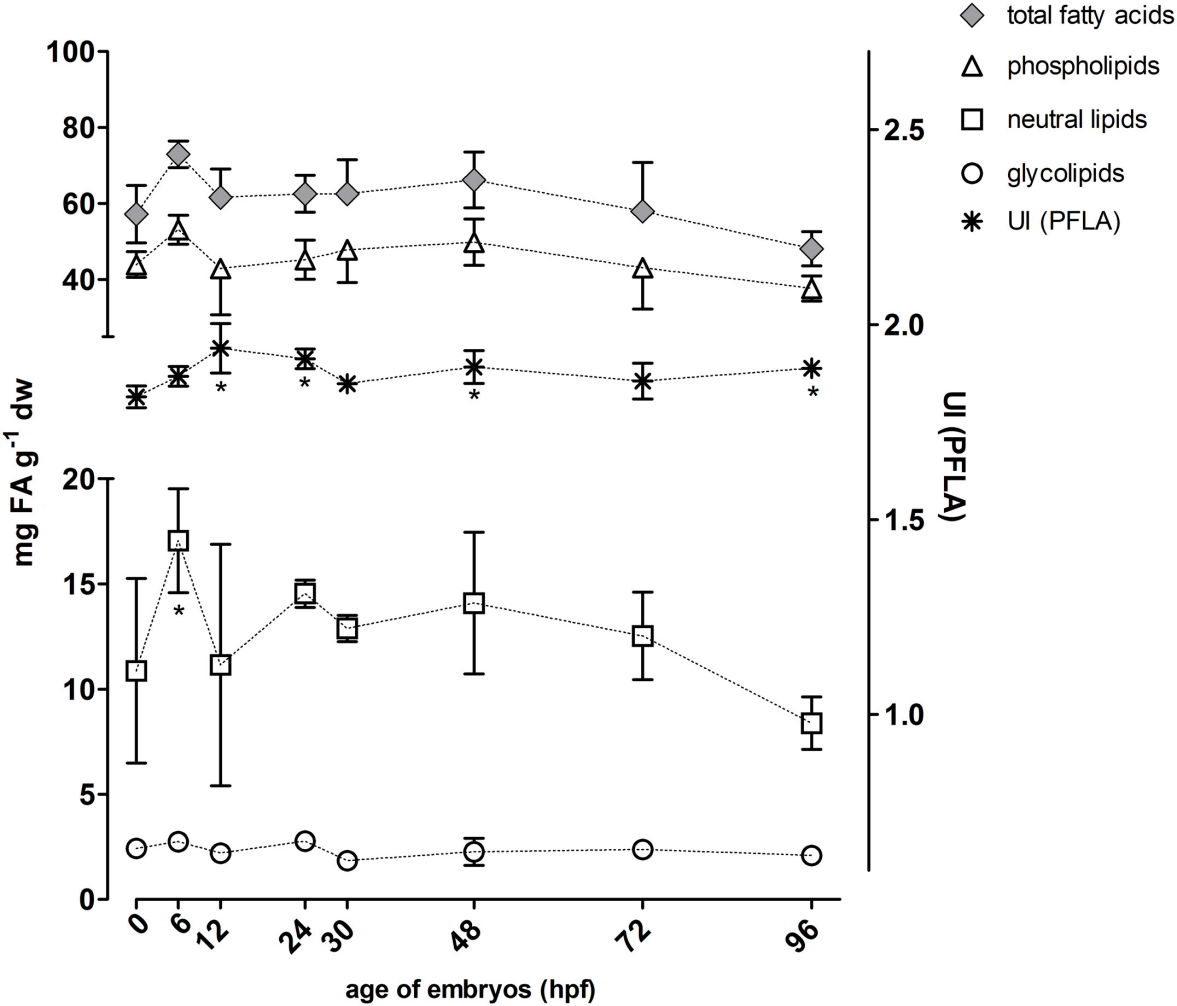
Quantities of different fatty acids (scaled to left y-axis) and the unsaturation index (UI) based on the phospholipids (PFLA) (scaled to right y-axis) for eggs/hatched embryos at 0–96 hpf. Total fatty acids, phospholipids, glycolipids and neutral lipids were normalized to dry weight. Symbols were connected to visualize trends. *: p < 0.05 (Dunnett’s test with 0 hpf as reference). n = 3–4 with 50 pooled individuals per n; hpf: hours post fertilization. Note that the left y-axis is divided into two sections with different scales.

The contents of total fatty acids ranged between 2.9 and 4.9 μg per individual at different stages, and there was a decreasing trend for fatty acid contents with advancing embryonic development, although the differences were not statistically significant (p > 0.05, Dunnett’s test with 0 hpf as control group). When related to dry weight (dw), contents of total fatty acids were almost constant in the different developmental stages with values between 48 and 73 mg g^-1^ dw (Figs 1B and 2).

#### Soluble protein

Levels of soluble protein varied between 19.3 and 24.9 μg per individual and showed an increasing trend up to 48 hpf when the protein level was significantly higher than at 0 hpf (p < 0.05, Fig 1A). When related to dry weight, the relative increase in soluble protein during embryo development becomes even more evident. Values ranged between 240 and 400 mg g^-1^ dw and peaked at 48 hpf (Fig 1B). From 30 hpf on, relative protein levels were statistically significantly higher than at 0 hpf (p < 0.05) (Fig 1B).

#### Carbon and nitrogen

Values measured for total carbon were between 27 and 39 μg per individual (Fig 1A). Carbon levels showed a continuous decrease throughout the considered developmental phase and were approximately 23% lower in 96 hpf embryos than at 0 hpf, paralleling the decrease of dry weight (Fig 1A). At 48, 72 and 96 hpf carbon levels were significantly lower than at 0 hpf (p < 0.05, Fig 1A). Levels of total nitrogen ranged between 5.9 and 7.9 μg per individual (Fig 1A). In comparison to carbon, nitrogen levels in 0 to 96 hpf embryonic stages showed more subtle changes with an overall decrease of only approximately 11% (Fig 1A). Only at 72 hpf, nitrogen levels were significantly lower than at 0 hpf (p < 0.05, Fig 1A). When related to dry weight, carbon and nitrogen levels did not show a stage-related trend, indicating that the decrease in dry weight over the considered developmental period was accompanied by corresponding losses of carbon and nitrogen (Fig 1B). Relative levels varied between 460 and 540 mg g^-1^ dw for carbon and between 97 and 111 mg g^-1^ dw for nitrogen; for neither element differences from 0 hpf were statistically significant (p > 0.05, Fig 1B).

#### Chorion

For determining to which extent the egg chorion in stages prior to hatching has an impact on dry weight and contents of protein, carbon and nitrogen, these parameters were compared for intact eggs and dechorionated embryos at 24, 30 and 48 hpf (refer to values for untreated eggs in Table 1). Lipids were not included in this analysis as the chorion consists mainly of glycoproteins whereas lipids are absent [23,24]. For dry weight and carbon and nitrogen contents, we found similar, stage-dependent patterns. For all three parameters, the values for intact eggs were statistically significantly higher than those for dechorionated embryos at 24 hpf (p < 0.05, t-test) but the differences became smaller in the more advanced stages (Table 1, values for untreated eggs). For the three parameters, the differences between intact eggs and dechorionated embryos ranged between 41–43% at 24 hpf, 25–28% at 30 hpf and 14–19% at 48 hpf. Whereas the values for intact eggs remained relatively constant between 24 and 48 hpf, values for dechorionated embryos increased by 28%, 32% and 24% for dry weight, carbon and nitrogen, respectively, thus approaching the values for intact eggs (Table 1, values for untreated eggs). This indicates that transfer of biological material from the chorion to the embryo tissue occurred in the considered developmental phase.

**Table 1:** Mean values for dry weight and the amounts of protein, carbon and nitrogen (± STD) for intact zebrafish eggs and embryos without chorion at 24, 30 and 48 hpf. Values are listed for untreated embryos and embryos that had been exposed to 2,4-DNP. Values for dechorionated embryos that were statistically significantly different from eggs with chorion from respective treatments (2,4-DNP exposure or control) are marked with * (p < 0.05; Student’s t-test). Note that values for untreated intact eggs correspond to respective data presented in Fig 1 and were listed here for comparison. For dry weight, carbon and nitrogen: n = 4 with 5 pooled individuals per n; for proteins: n = 5–7 with 30 pooled individuals per n; hpf: hours post fertilization.

		dry weight		protein		carbon		nitrogen	
*μg/individual*		intact egg	dechor. embryo	intact egg	dechor. embryo	intact egg	dechor. embryo	intact egg	dechor. embryo
untreated	24 hpf	69.5 ± 4.1	39.5 ± 15.5*	21.3 ± 3.4	22.0 ± 0.8*	33.5 ± 1.6	19.7 ± 7.4*	6.9 ± 0.3	4.1 ± 1.6*
	30 hpf	67.0 ± 8.3	50.5 ± 12.6	23.6 ± 1.9	22.6 ± 0.5	33.6 ± 4.3	24.0 ± 5.2	6.9 ± 0.9	5.0 ± 1.1
	48 hpf	62.0 ± 8.4	50.5 ± 6.7	24.9 ± 2.2	23.5 ± 1.2	29.4 ± 3.4	24.4 ± 3.1	6.2 ± 0.7	5.3 ± 0.6
2,4-DNP treated	24 hpf	65.0 ± 10.1	41.3 ± 7.6*	22.4 ± 1.0	21.8 ± 1.3	33.9 ± 4.9	21.4 ± 4.5*	7.4 ± 1.3	4.2 ± 0.9*
	30 hpf	67.0 ± 3.0	55.0 ± 10.1	23.6 ± 1.1	22.8 ± 0.8	34.5 ± 1.1	26.3 ± 5.3	8.5 ± 0.6	5.2 ± 1.1*
	48 hpf	74.0 ± 5.8	58.5 ± 9.6	24.4 ± 1.0	24.3 ± 1.2	34.5 ± 2.9	27.4 ± 3.8*	7.1 ± 0.6	5.5 ± 0.8*

### Fatty acid composition

#### Fatty acid fractions

Values related to dry weight for total fatty acids, phospholipids, neutral lipids and glycolipids for 0–96 hpf embryonic stages are depicted in Fig 2. Overall, the phospholipid fatty acid (PLFA) fraction accounted for 69–78% of the total fatty acids followed by neutral lipid fatty acids accounting for 17–23% and glycolipid fatty acids accounting for 2.9–4.4% of total fatty acids. Whereas values for PFLAs and glycolipids were relatively constant in 0–96 hpf embryonic stages, neutral lipid values showed a slight decrease from 48 hpf on, which, however, was not significant compared to 0 hpf (p > 0; Fig 2). Phospholipids and neutral lipids showed a peak at 6 hpf (Fig 2) that however was attributed to the low dry weight value at this stage (Fig 1a). In more advanced embryonic stages, total fatty acids tended to decrease, in parallel to neutral lipids and PLFAs (Fig 2).

#### Phospholipid fatty acids

**Fig 3:**
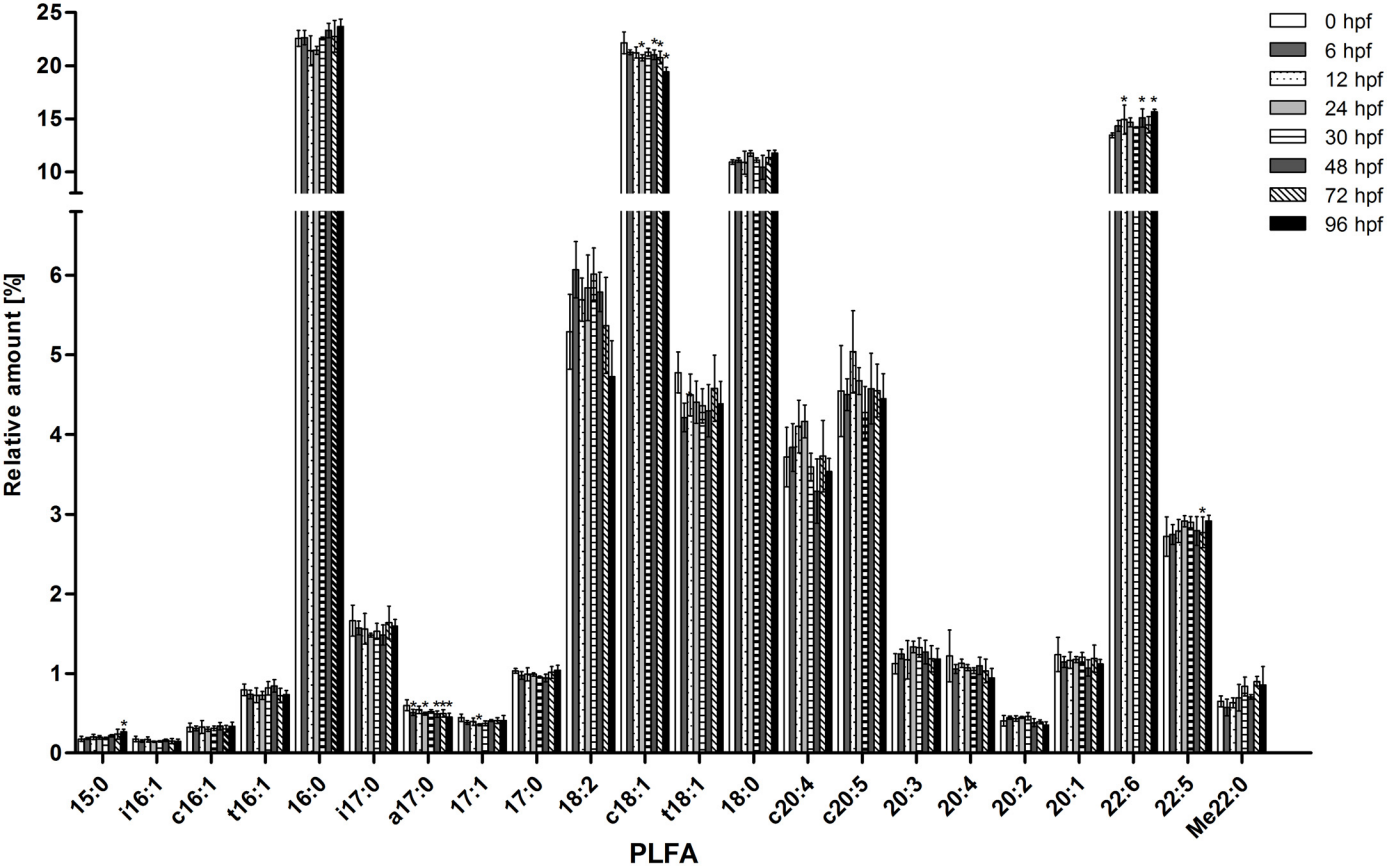
Relative amounts of phospholipid fatty acid (PLFA) fractions in eggs/hatched embryos of the zebrafish at 0–96 hpf (one bar for each stage). n = 3–4 with 50 pooled embryos per n; hpf: hours post fertilization. Note that the y-axis is divided into two sections with different scales.

The PLFAs constituting the largest fatty acid fraction were further analyzed in more detail and the relative contribution of the different PLFAs was determined (Fig 3). The saturated palmitic acid (C16:0; 21–24%) and the monounsaturated oleic acid (C18:1*cis*Δ^9^; 19–22%) were found to be the dominating fatty acids in this fraction of total PLFAs. The polyunsaturated docosahexaenoic acid (C22:6) accounted for 13–16% of total PLFAs, followed by the saturated stearic acid (C18:0; 10–12%). The relative amounts of all other identified fatty acids were less than 6% of total PLFAs. The PLFA pattern was relatively constant between 0–96 hpf and statistically significant differences between more advanced stages and 0 hpf were only seen in few cases (p < 0.05, Fig 3). For example, the percentage of a17:0 was significantly lower than at 0 hpf from 6 hpf onwards, and the percentage of c18:1 was significantly lower than at 0 hpf from 24 hpf up to 96 hpf (except for 30 hpf). In the case of 17:1, the percentage was lowest at 24 hpf; this value is significantly lower than the one at 0 hpf. Other PLFAs, such as 15:0, 22:6 and Me22:0 increased during embryonic development. In the case of 15:0, this increase is statistically significant compared to 0 hpf only at 96 hpf (p < 0.05); the percentage of 22:6 was significantly higher at 12, 48 and 96 hpf compared to 0 hpf (p < 0.05); for the percentage of Me22:0 this was the case at 72 hpf. Despite these changes for single PFLAs, the general PLFA pattern was overall constant over the different developmental stages.

In addition to determining individual PLFAs, the unsaturation index (UI) based on the relative abundance of PLFAs (see below) was calculated (Fig 2). The UI is a condensed index for membrane fatty acid composition and serves as measure for the control of membrane fluidity by adjusting the fatty acid composition of the membrane [25]. Although variations in UI were within a relatively narrow range with values between 1.81 and 1.94, a slight increase in UI values was seen in the first 12 hrs. UI values at 12, 24, 48 and 96 hpf were significantly higher than at 0 hpf (p < 0.05, Fig 2).

### Effects of 2,4-DNP exposure

To test the effect of 2,4-DNP on the body mass parameters during embryonic development, 2,4-DNP was applied at a concentration corresponding to the LC10 (48 h) for zebrafish embryos [26], for which we assumed strong sublethal effects. All parameters were examined at 24, 30 and 48 hpf. When compared to untreated eggs at the respective stages, statistically significant effects were only found for lipids (p < 0.05, Student’s t-test; Table 2), but not for dry weight and protein, carbon and nitrogen contents (p > 0.05, values for these parameters for 2,4-DNP treated eggs are listed in Table 1). However, 2,4-DNP effects on embryos with respect to carbon and nitrogen levels became obvious when comparing intact eggs and dechorionated embryos. Without chemical treatment, significant differences between intact eggs and dechorionated embryos were only seen at 24 hpf, but not in the later stages (see above, refer to data for untreated eggs in Table 1). In contrast, upon treatment with 2,4-DNP, carbon levels (per individual) were statistically significantly lower in dechorionated embryos than in intact eggs not only at 24 but also at 48 hpf and nitrogen levels were significantly lower in dechorionated eggs at all investigated stages (24, 30, and 96 hpf; p < 0.05; Table 1).

**Table 2:** Mean values (± STD) for contents of total fatty acids and lipid fractions as well as unsaturation indices (UI) of the PLFA fraction in untreated zebrafish eggs and eggs treated with 2,4-DNP at 24, 30 and 48 hpf. Values for 2,4-DNP treated eggs that were statistically significantly different from those for untreated eggs at the respective stage are marked with * (p < 0.05; Student’s t-test). Note that values for untreated eggs correspond to respective data presented in Fig 2 and are listed here for comparison. n = 3–4, with 50 eggs per n; hpf: hours post fertilization.

	total fatty acids (*mg g* ^*-1*^ *dw*)		PFLAs (*mg g* ^*-1*^ *dw*)		glycolipids (*mg g* ^*-1*^ *dw*)		neutral lipids (*mg g* ^*-1*^ *dw*)		PFLA UI	
	untreated	2,4-DNP	untreated	2,4-DNP	untreated	2,4-DNP	untreated	2,4-DNP	untreated	2,4-DNP
24 hpf	62.5 ± 4.9	50.4 ± 11.6	45.2 ± 5.2	38.7 ± 4.9	2.8 ± 0.2	1.0 ± 0.5*	14.5 ± 0.6	14.3 ± 2.4	1.91 ± 0.02	1.48 ± 0.02*
30 hpf	62.6 ± 9.0	47.8 ± 3.7*	47.8 ± 8.7	33.5 ± 1.0*	1.8 ± 0.3	0.7 ± 0.1*	12.9 ± 0.6	13.7 ± 2.7	1.85 ± 0.01	1.44 ± 0.03*
48 hpf	66.2 ± 7.4	51.6 ± 7.0*	49.9 ± 6.1	32.6 ± 11.7*	2.3 ± 0.7	0.9 ± 0.5*	14.1 ± 3.4	18.0 ± 7.6	1.89 ± 0.04	1.76 ± 0.05*

The relative amounts of total lipids were significantly lower in the 2,4-DNP treatments at 24 hpf (25% lower) and at 30 and 48 hpf (about 35% lower at each stage; p < 0.05 for all stages; Table 2). These differences in total fatty acids can be assigned to changes caused by 2,4-DNP in the PFLA and the glycolipid fatty acid fractions; neutral lipid fatty acid levels were not significantly different between 2,4-DNP exposed and untreated eggs (p > 0.05, Table 2). The largest differences between 2,4-DNP treated and untreated eggs were seen for glycolipids. Relative glycolipid contents decreased by 72% at 24 hpf, by 64% at 30 hpf and by 59% at 48 hpf in 2,4-DNP treatments (p < 0.05 for all stages, Table 2) showing narrowing differences between treated and untreated eggs over time. In contrast, differences between 2,4-DNP treated and untreated eggs in relative PFLA contents constantly increased between 24 and 48 hpf. PFLA values in the 2,4-DNP treatments were decreased by 14% at 24 hpf, by 30% at 30 hpf and by 45% at 48 hpf compared to untreated eggs (p < 0.05 for all stages, Table 2).

**Fig 4:**
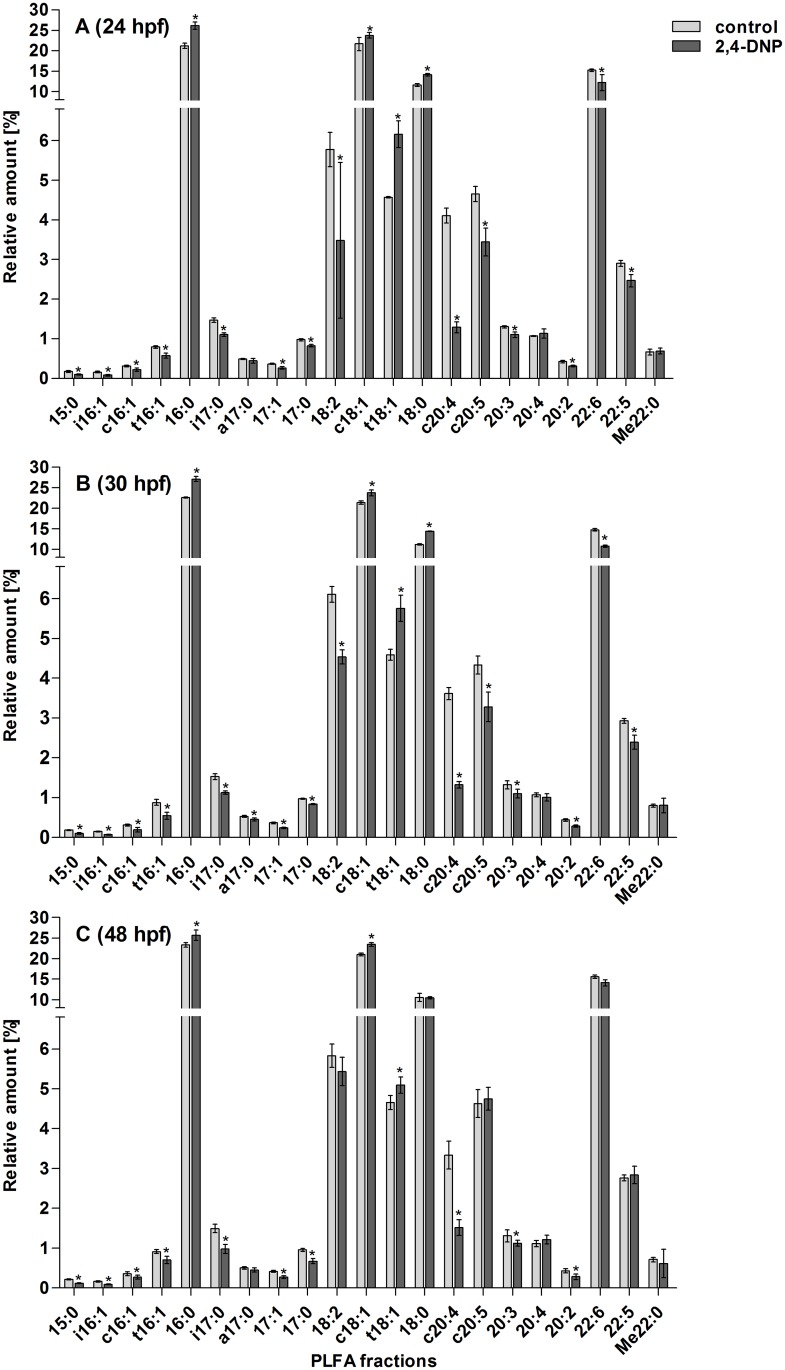
PLFA patterns of untreated (light grey) and 2,4-DNP-treated (dark grey) embryos at 24 (A), 30 (B) and 48 hpf (C). * indicates significant differences between untreated and 2,4-DNP-treated embryos (p < 0.05; Student's t-test). n = 3–4 with 50 pooled embryos per n. Note that the y-axes are divided into two sections with different scales.

The relative contents of 15 out of the 21 individual PFLAs detected were lower in 2,4-DNP treated than in untreated eggs, four (16:0, c18:1, t18:1 and 18:0) were higher and two (20:4 and Me22:0) showed no trend (Fig 4). For most PLFAs, these differences were observed in all examined embryonic stages; for five PLFAs (18:2, 18:0, c20:5, 22:6, 22:5) significant differences (p < 0.05, Student’s t-test) were seen at 24 and 30 hpf, but not at 48 hpf; in case of a17:0, the relative contents were significantly lower only at 30 hpf. 22:6 was the only highly abundant PLFA (>10% of total PLFA) which decreased in relative abundance due to treatment with 2,4-DNP. The relative abundance of all other highly abundant PLFAs (16:0, c18:1, t18:1 and 18:0) increased upon exposure to 2,4-DNP. In the case of 18:0, this increase was statistically significant only at 24 and 30 hpf, but not at 48 hpf (Fig 4). Thus, overall more differences occurred between 2,4-DNP treated and untreated eggs at 24 and 30 hpf than at 48 hpf.

The changes in PFLAs upon 2,4-DNP treatment are also reflected by UI values that were significantly lower in 2,4-DNP treatments than in untreated eggs (p < 0.05 for all stages, Student’s t-test, Table 2). As for PFLA patterns, differences between 2,4-DNP treated and non-treated eggs were larger at 24 and 30 hpf than at 48 hpf. Thus, at 24 and 30 hpf, UI values were around 1.5 in 2,4-DNP treated eggs compared to 1.9 in non-treated eggs; at 48 hpf the difference was only 0.1 (Table 2).

## Discussion

Within the four days period considered here, zebrafish embryos undergo extreme morphological changes from the freshly fertilized egg consisting almost exclusively of yolk encased by a chorion to a freely swimming organism with highly differentiated tissues and cells and with only little yolk reserves remaining [27]. During this developmental phase, the embryos do not take up nutrients; the differentiation and developmental processes are entirely fueled by endogenous reserves. Our results show that in spite of these fundamental morphological changes, the parameters examined here changed only relatively little over time. During development, dry weights of eggs/hatched embryos decreased with tissue contents of carbon, nitrogen and lipids also following a decreasing trend, whereas, interestingly, protein contents increased between 0–96 hpf. Upon exposure of embryos to 2,4-DNP, major changes were seen for PFLA and glycolipids, but not for the other examined parameters.

### Protein/lipid and carbon/nitrogen tissue levels in relation to dry weights

Data for the here measured body mass parameters have been determined for a range of fish species (for an overview, refer for instance to Kamler (2008) and Watanabe and Kiron (1994) [28,29]). Series of embryonic/larval teleost stages were also examined [29,30] but to our knowledge we present the first such data for zebrafish, which is an important model for ecotoxicological, pharmaceutical and developmental studies.

For zebrafish embryos prior to hatching, values of 63.2 μg for dry weight and of 6.5 μg for lipid per egg (corresponding to 10.3% of dry weight) were previously published [31]. The value for dry weight approximately corresponds to the values for eggs at 30 and 48 hpf reported here (Table 1) which may be seen as indication that dry weights of eggs of cultivated zebrafish are relatively uniform across strains and labs. For seven days old zebrafish, dry weights of 39.1 μg per individual were reported [31] indicating that dry weight continues to decrease after 96 hpf. In the same study, 6.5 ± 1.3 μg per individual were reported for the lipid content in zebrafish eggs [31], which is about 30% higher than the values we found at 30 and 48 hpf embryos (Table 1); these differences in results may be due to differences in the applied methods for quantification of lipids in the two studies. However, overall, lipid contents of zebrafish eggs appear to be low when compared to eggs from other teleost species with lipid content levels that in some cases reach 30% of dry weight [28,32,33].

Lipids and proteins are main tissue components, but in sum amounted to only one third to one half of the tissue dry weights at the respective stages in our study. Protein levels of the different embryo stages estimated based on nitrogen contents according to Jones (1931) [34] (% nitrogen (dw) × 6.25) are 1.2–2.3 times above the levels that we determined with the Lowry assay and it therefore may be assumed that the total amount of protein was underestimated with our assay. The chromogenic amino acids are mainly tyrosine and tryptophan and, to a lesser extent, cystine, cysteine, and histidine; thus, differing amino acid compositions of the protein standard BSA and the proteins in the sample can result in erroneous protein quantification [35]. Vitellogenin (Vtg) is quantitatively the predominant protein in fish embryos that should therefore majorly contribute to the colorimetric signal. We therefore examined, whether the use of BSA as protein standard could result in errors in protein quantification in the embryos. When comparing amino acid sequences of BSA (NCBI accession no. 280717) and different zebrafish Vtg isoforms (Vtg 1–7; NCBI acc. nos. 559475, 559931, 30518, 678536, 64260, 559229, 559856) we found that percentages of tyrosine and tryptophan in BSA and Vtgs were similar (Tyr: 3.5% in BSA, 2.5–3.4% in different Vtg isoforms; Trp: 0.5% in BSA, 0.6–1.4% in different Vtg isoforms). Thus, it seems unlikely that the use of BSA as protein standard resulted in underestimation of proteins in the fish embryos. Fish embryos contain free amino acids (2–5% of amino acids are free amino acids in embryos of fresh water fish [36]) which may contribute to the discrepancy between our measured protein levels and the estimated values based on nitrogen, if the abundance of chromogenic amino acids among the free amino acids is low. A further possibility is that part of the protein in the egg/embryo was not solubilized in the protein extraction buffer and was therefore not detected with the Lowry assay leading to underestimation of the entire protein content. Indication for this comes from the comparison of the nitrogen and protein data for intact eggs and dechorionated embryos at 24, 30 and 48 hpf. Whereas nitrogen contents remained similar in intact eggs of all three stages, there was a continuous increase of nitrogen in dechorionated embryos between 24 and 48 hpf of approximately 30% overall (Table 1). The embryos that were analyzed without chorion were dechorionated briefly prior to freezing and thus development of those embryos up to the time point of sampling had proceeded within the chorion; it hence appears that nitrogen transgressed from the chorion to the embryo tissue as long as the embryo was within the chorion. In contrast, in intact eggs, with embryo and chorion together, the overall nitrogen balance remained stable over the three examined stages. Simultaneously to the increase of nitrogen levels in the embryo tissue, protein contents were also becoming higher (Table 1) indicating transgression of nitrogen as protein to the embryo tissue. Interestingly, increases in tissue protein content from 24 to 48 hpf were seen in both dechorionated embryos and intact eggs (Table 1). We assume that due to its rigid structure, the protein in the chorion (mainly glycoproteins) is not accessible to detection via the Lowry assay with our protein extraction protocol as it may not be solubilized under these conditions; however, the proteolytic activity leading to chorion softening prior to hatching [37] may lead to the release of digested protein from the chorion that is then taken up by the embryo, where it is present in a form that is solubilized with protein extraction.

Apart from lipids and proteins, carbohydrates may also considerably contribute to the embryo body mass. Carbohydrates in the form of glycogen only play a minor role in the nutrition of the unhatched embryo [36], but glycosyl components of Vtg [38] may be abundant.

Carbon and nitrogen levels of the eggs/hatched embryos amounted to 44–52% and 8–13% of respective dry weights, respectively, in sum corresponding to 50–60% of dry weights, which is in agreement with values reported for these elements in teleosts of different species and at different developmental stages [39–43]. Although so far not quantified in fish, further elements in general majorly contributing to body mass are oxygen, hydrogen and sulfur [44].

### Changes of the measured parameters during embryonic development

Decreases in dry weights and the tissue contents of lipids, carbon and nitrogen over the considered developmental phase are associated with metabolic activity and respiration leading to losses in body mass that are not compensated by nutrient uptake. Respiration in zebrafish embryos linearly increases with development; oxygen consumption almost doubles between 24 and 48 hpf from approximately 60 to almost 120 pmol O_2_ min^-1^ embryo^-1^ [45]. In return to oxygen uptake, carbon dioxide is released and this loss of carbon via carbon dioxide can be regarded the reason for the observed decrease in embryo tissue carbon levels and consequently in dry weight. Respiration rates for zebrafish embryos reported by Stackley et al. (2011) [45] are up to 48 hpf but assuming that respiration continues to increase by the same rate as between 0 and 48 hpf we calculated an overall uptake of approximately 700 nmol oxygen by zebrafish embryos between 0 and 96 hpf. Assuming a respiratory quotient (RQ) of 1 this in turn equals the overall release of 700 nmol (8.4 μg) carbon as carbon dioxide by the embryos; this total amount of released carbon is almost identical to the overall decrease of carbon levels by 8.7 μg between 0 and 96 hpf resulting from our carbon quantifications (Fig 1). Lipids, free amino acids and proteins are the main nutritional components for fish embryos prior to feeding [36] and accordingly the constant decrease in lipids observed here can be seen as the result of catabolic activity. Similarly, in the context of protein catabolism, the decrease in nitrogen levels can be associated with the active excretion of ammonia and urea that has been quantified in zebrafish embryos [46]. Protein levels that increase with development (Fig 1) seem to contradict this interpretation; however, as outlined in the previous section, the protein levels as indicated by the Lowry assay may not be the entire but the soluble protein.

### Fatty acid profiles

In embryonic stages of teleosts, lipids are important components of Vtgs, large multidomain apolipoproteins [38], and function as catabolic substrates [36] and as structural components of cellular membranes [47]. Phospholipids, predominant lipids in animal cellular membranes [48], represented the largest lipid fraction in teleost embryos in this and in other studies, followed by neutral lipids [32,49–51]. The slight decrease in PFLA contents during development that we observed (Fig 2) are in accordance with a specific role of PFLAs as nutrient source for teleost embryos [51–53]. Neutral lipids, generally serving as storage fat, were found to be present as droplets within the yolk of zebrafish embryos [54]; they serve as nutrient source for the developing embryo and, in accordance with progressively decreasing contents from 48 hpf on in our study (Fig 2), are consumed together with the yolk supply [54]. Further, glycolipids, by overall content a minor lipid fraction, are, as cellular membrane components, important for anchoring/sorting of membrane structures and signaling/signal transduction [55] but they are also present as droplets in the yolk of teleost eggs [56]. As the other lipid fractions, glycolipids decreased along with dw in developing fish embryos in our study (Fig 2) suggesting as well a role in embryo nutrition.

Overall, despite changes in abundances of some PFLAs, PFLA profiles were similar across different zebrafish embryo stages (Fig 3). In all stages, most abundant PFLAs (15–25%) were 16:0, c18:1, 18:0 and 22:6 (Fig 3). This generally corresponds to PFLA patterns found in eggs of other teleost species [30,32,50,57–59] with exception of 18:0 that was low (2.1–3.5%) in eggs of marine fish species [30,50,58].

The slightly increasing UI values reflect higher abundance of unsaturated PFLAs with development which can be seen as indication for a trend towards an elevation of cell membrane fluidity. As overall membrane fluidity is not expected to change, alterations in the PFLA UI may point to shifts in abundances of membrane components that affect membrane fluidity. Thus, modification of the PFLA UI can be seen as adaptive response to the increased occurrence of components that elevate membrane viscosity [60].

### Effects of 2,4-DNP exposure

Interference of 2,4-DNP with energy metabolism [21] and with lipid synthesis [61] are indicated by profound 2,4-DNP effects on PFLA and glycolipid contents. Low PFLA and glycolipid levels in 2,4-DNP-exposed embryos indicated enhanced depletion of lipids by increased lipid catabolism. Interestingly, levels of neutral fatty acids were not affected by 2,4-DNP exposure but it is not clear, why catabolism of neutral fatty acids that serve as storage lipids was not enhanced by 2,4-DNP. A possible explanation may be that neutral fatty acids are not yet available for catabolism for the embryos at the examined stages (24–48 hpf). Thus, in the time series with embryos not exposed to a chemical neutral lipid levels clearly decreased only after 48 hpf (Fig 2) suggesting that before 48 hpf neutral lipids were not yet catabolized.

Apart from 2,4-DNP interference with the embryos’ energy metabolism the clear effects on the PFLA patterns (Fig 4) also indicate 2,4-DNP interactions with cellular membranes. The compound 2,4-DNP is slightly lipophilic (logP_octanol/water_ = 1.7 [62]) and is able to accumulate in cellular membranes resulting in an increase in membrane fluidity [22,63]. The changes of the PFLA pattern in zebrafish embryos upon exposure to the chemical indicate an adaptive response against the changes in membrane fluidity, a process called “homeoviscous adaptation” [64]. The changes in the PFLA patterns of 2,4-DNP-exposed zebrafish embryos generally comprised a decrease of highly unsaturated and branched PLFAs and an increase of saturated straight chain PLFAs. Such shifts in the relative distribution of polyunsaturated towards higher amounts of saturated fatty acids increase the cellular membrane’s viscosity [65]. This shift in fatty acid distribution towards higher viscosity in 2,4-DNP-exposed zebrafish embryos is reflected by decreasing UI values (Table 2). The fatty acid composition of cellular membranes of teleosts has already been studied in the context of temperature dependence of membrane viscosity and membrane fatty acid compositions have been found to vary across different teleost species as adaptations to the ambient temperature regimes at which the teleost species live [66–68]. Further, indications were obtained that compositions of cellular membranes of fish embryos raised at different temperatures are adapted in order to maintain membrane fluidity at a respective temperature [58,59]. Our results show that likewise the cellular membrane composition of zebrafish embryos is modified as adaptive response to chemically caused changes in membrane fluidity.

Although not quantified by other means, our data, in addition to effects on lipid catabolism and on membrane lipid fluidity, may also indicate retardation of embryo development as a result of 2,4-DNP exposure. Thus, as discussed above, comparisons of carbon and nitrogen levels of intact eggs and dechorionated embryos at 24, 30 and 48 hpf indicate transition of material from the chorion to the embryo in that time period. This, however, was only seen in individuals kept under control conditions but not in 2,4-DNP treatments (Table 1). As development of surviving embryos from 2,4-DNP exposures proceeded overall normally, we assume that the process of transition of biological material observed in the control embryos was delayed by several hours.

### Conclusions

Our data on body mass related parameters in developing zebrafish embryos overall reflect the consumption of yolk reserves as energy source. Those data may serve as basis to determine the energy costs of zebrafish embryo development. Further, they may be useful as basis for assessing bioaccumulation of chemicals in zebrafish embryos based on physicochemical parameters. Effects by the test compound 2,4-DNP on lipids in zebrafish embryos were expected, however, particularly the pronounced 2,4-DNP effects on the PFLA pattern suggest that this may be a useful endpoint to study membrane effects of lipophilic chemicals.

## Methods

### Culture conditions of zebrafish, collection of eggs and cultivation of embryos

The study was performed with eggs and hatched embryos of the zebrafish, *Danio rerio* (Hamilton-Buchanan, 1822). Adult zebrafish were obtained from a local supplier and were maintained and bred according to standard protocols [69]. Collection of eggs and cultivation of embryos were performed as described elsewhere [7,13]. In short, fertilized eggs were separated from unfertilized eggs and incubated at 27°C ±1 in embryo culture water according to OECD (2013) [13]. Embryos at 0.5, 6, 12, 24, 30, 48, 72 and 96 hours post fertilization (hpf) were flash frozen in liquid nitrogen and stored at -80°C for a maximum of 20 days until analysis. Depending on the analytical procedure, 5–50 individuals were pooled for each sample. Experiments were performed with four to seven independent replicates.

### Exposure experiments with 2,4-dinitrophenol

The exposure solution contained 14 μM of 2,4-dinitrophenol (2,4-DNP; CAS 51-28-5 Fluka) corresponding to the LC10(48h) for zebrafish embryos [26]. Freshly fertilized zebrafish eggs were added to 0.7mL of exposure medium per egg and incubated for 24, 30 and 48 h, along with controls containing no 2,4-DNP. After exposure, eggs were flash frozen in liquid nitrogen and stored at -80°C for a maximum of 20 days until analysis.

### Determination of dry weight, carbon and nitrogen contents

Five eggs or dechorionated or hatched embryos per stage were transferred to tin capsules (8 x 5 mm, Art. # 201050, LabNeed, Germany) along with 100μL of Milli-Q water. The number of eggs or embryos in each sample corresponded to a dry weight of at least 100 μg for elemental analyses. The samples were dried at 60°C for 42 hrs and dry weights were determined with a micro balance (Sartorius ME235S). The amounts of carbon and nitrogen in the tissue were quantified with an elemental analyser (EURO EA3000 CN-Elemental Analyser, EuroVector). Helium at a flow rate of 124mL/min served as carrier gas. Samples were oxidized at 1010°C in a reactor containing chromium oxide and silvered cobaltous-cobaltic oxide as catalysts. The resulting gases were then reduced in a reactor containing copper to obtain CO_2_ and N_2_. The separation of gases was performed at 70°C by gas chromatography. The system was equipped with a thermal conductivity detector. Acetanilide (CAS 103-84-4, Hekatech) served as external standard. C and N element contents of tissues were quantified based on peak areas.

### Quantification of protein contents of different zebrafish embryo stages

Thirty pooled eggs or dechorionated or hatched embryos per stage were homogenized on ice in 600 μL of phosphate buffer (100mM, pH 7.5) for 15 seconds with a Ultra Turrax device. The homogenates were centrifuged for 15min at 10,000x g at 4°C. Protein contents of fish embryos were determined according to the method by Ellman (1961) [70] adapted to microtiter plates [71]. Briefly, 5 μL of supernatants of each sample were analyzed in quadruplicates using the DC Protein Assay (BioRad) according to the manufacturer’s instructions. Bovine serum albumine, fraction V (BSA; CAS 9048-46-8, SERVA) was used as external standard protein. Absorption was measured with a spectrophotometer at 750 nm and converted to protein concentrations via a calibration curve ranging from 1 to 8mg/L BSA protein. For each developmental stage five to seven replicates were analyzed. The protein amounts of all replicates for one embryonic stage were averaged and standard deviations were calculated.

### Lipid extraction, separation and derivatization to FAME

Eggs/hatched embryos were harvested at 0–2, 6, 12, 24, 30, 48, 72 and 96h post fertilization (hpf) and pools of 30 individuals per life stage were immediately frozen at -80°C in 1.5mL Eppendorf reaction tubes and stored for a maximum of one week prior to extraction. Extraction of hydrophobic tissue components was based on the protocol published in [72]. For improved lipid extraction, we introduced an additional step for disrupting eggs/embryos using a FastPrep bead mill. Frozen eggs/embryos were suspended in 1mL potassium phosphate buffer (K_2_HPO_4_, 0.05M, pH 7) and transferred to a FastPrep tube (MP Biomedicals, Lysin Matrix E, 2mL, Ref. 6914-100). For tissue homogenization, the FastPrep mill (MP Biomedicals, FastPrep-24) was operated at speed 6 for 30sec. The entire content of each FastPrep tube was transferred to a 15mL glass vial with 1mL potassium phosphate buffer. Each FastPrep tube was repeatedly washed with 4 × 1mL methanol, which was transferred to the 15mL glass vial. One additionalmL methanol and 2.5mL chloroform were added to the suspension, which was shaken for 2h at 350rpm on an orbital shaker at room temperature. Subsequently, 2.5mL distilled water and 2.5mL chloroform were added and samples were left at 5°C for 18h for phase separation. The entire chloroform phase was transferred to a clean glass vial and the solvent was evaporated under a stream of nitrogen. The dried hydrophobic components were dissolved in 0.2mL chloroform.

Separation of different lipid classes was performed by passage of the samples over approximately 0.5 g silica gel (Unisil activated silicic acid; Clarkson Chromatography Products Inc., South Williamsport, PA, USA) in a glass column (BAKERBOND SPE; 8mL; J.T.Baker, Deventer, Netherlands) containing a PTFE disc. Prior to addition of the sample, the packed column was conditioned by successively washing with 5 mL ammonium acetate (0.02M in methanol), 5 mL acetone and 5 mL chloroform. Neutral lipids were eluted from the silica gel with 10 mL chloroform, glycolipids with 10 mL acetone and phospholipids with 10 mL methanol. The fractions were collected separately in glass vials and dried under a gentle stream of nitrogen gas.

The fatty acids in the three lipid fractions were derivatized to fatty acid methyl esters (FAME) according to Morrison and Smith (1964) [73]. Methylation was carried out with 1.2mL boron trifluoride methanol complex (20% in methanol; VWR, Darmstadt, Germany) at 95°C for 15min. The reaction was stopped by adding 0.6 mL distilled water and 1mL n-hexane (HPLC grade, VWR, Darmstadt, Germany). After 60 sec shaking (Vortex-Genie 2 Shaker), an aliquot of 0.5 mL was taken from the hexane phase of each sample. The solvent was evaporated under a gentle stream of nitrogen and the dried sample was dissolved in 0.5 mL n-hexane including internal standard (10 μg/mL methyl heneisosanoate [21:0]; Sigma Aldrich, Munich, Germany). Samples were stored at 5°C until analysis by gas chromatography/mass spectrometry (GC/MS).

### GC-MS analysis and determination of the degree of saturation and the unsaturation index (UI)

FAME samples were analyzed by GC/MS using a gas chromatograph (7890A GC system, Agilent Technologies, Waldbronn, Germany) coupled to a mass spectrometer (MS, 5975C inert MSD with triple axis detector, Agilent Technologies) and equipped with an autosampler (7693, Agilent Technologies) and a split/split-less injector (G4513A, Agilent Technologies). The column was a BPX5 capillary column (30 m × 0.25mm × 0.25 μm; SGE, Kiln Farm Milton Keynes, UK), and helium at a flow rate of 1.2 mL min^-1^ was used as carrier gas. The injection volume was 1 μl. The injection was splitless, the injector temperature was 280°C. The initial oven temperature was 50°C for 1min and the final temperature was 300°C with an increase of 4°C min^-1^ to 250°C and 20°C min^-1^ to 300°C followed by 5min isothermal conditions. The temperature of the transfer line to the MS was set to 300°C. Ionization of the eluting compounds was performed by electron impact at 70eV. The ion source temperature was 230°C and the quadrupole temperature was 150°C. Full scans were acquired from m/z 40 to m/z 500. Data acquisition was performed using the MSD-Chemstation Software (MSD ChemStation E.02.00.493; Agilent Technologies). Fatty acids were identified by comparison of the retention times of the peaks with those of a commercial qualitative standard of bacterial acid methyl esters (BAME, Sigma Aldrich) and of the mass spectra with the NIST data base using the NIST Mass Spectral Search Programm (NIST MS Search 2.0f). The internal standard of known concentration allowed the quantification of the fatty acids based on integration of the peak areas.

The relative amounts of the individual fatty acids were used to calculate the unsaturation index (UI) as follows [25,74]:

UI =[(% 16:1+18:1)+(% 18:2×2)+(% 18:3×3)+ …]/100

### Statistical analysis

Normal distribution of data was assumed and parametric statistics were used for data analyses. Mean values and standard deviations were determined from replicate values. For determining statistically significant changes of the examined parameters within the considered developmental period (0–96 hpf), ANOVA followed by Dunnett’s test with 0hpf as control group were performed. Student’s t-test was applied to compare dechorionated embryos and intact eggs and 2,4-DNP treatments with untreated eggs or dechorionated embryos from the respective stages. Differences were regarded significant if p < 0.05. Statistics were performed using the software JMP 10.0.0 (64-Bit-Version, SAS Institute GmbH, Germany).

### Ethics Statement

All zebrafish husbandry and experimental procedures were performed in accordance with the German animal protection standards and were approved by the Government of Saxony, Landesdirektion Leipzig, Germany (Aktenzeichen 75–9185.64) and were based on the guidelines on the protection of experimental animals by the Council of Europe, Directive 2010-63-EU, which allows zebrafish embryos to be used up to the moment of independent feeding (approximately 5 days after fertilization). The embryos used in experiments here were not older than four days and therefore no authorization was required by the local authority.
